# Reliability of a functional magnetic resonance imaging task of emotional conflict in healthy participants

**DOI:** 10.1002/hbm.24883

**Published:** 2019-12-03

**Authors:** Stefanie Hassel, Gulshan B. Sharma, Gésine L. Alders, Andrew D. Davis, Stephen R. Arnott, Benicio N. Frey, Geoffrey B. Hall, Jacqueline K. Harris, Raymond W. Lam, Roumen Milev, Daniel J. Müller, Susan Rotzinger, Mojdeh Zamyadi, Sidney H. Kennedy, Stephen C. Strother, Glenda M. MacQueen

**Affiliations:** ^1^ Department of Psychiatry, Cumming School of Medicine University of Calgary Calgary Alberta Canada; ^2^ Mathison Centre for Mental Health Research and Education University of Calgary Calgary Alberta Canada; ^3^ Graduate Program in Neuroscience McMaster University, and St. Joseph's Healthcare Hamilton Hamilton Ontario Canada; ^4^ Department of Psychiatry and Behavioural Neurosciences McMaster University Hamilton Ontario Canada; ^5^ Rotman Research Institute Toronto Ontario Canada; ^6^ Mood Disorders Program and Women's Health Concerns Clinic St. Joseph's Healthcare Hamilton Ontario Canada; ^7^ Department of Psychology, Neuroscience and Behaviour McMaster University Hamilton Ontario Canada; ^8^ Department of Computer Science University of Alberta Edmonton Alberta Canada; ^9^ Department of Psychiatry University of British Columbia Vancouver British Columbia Canada; ^10^ Department of Psychiatry Queen's University and Providence Care Hospital Kingston Ontario Canada; ^11^ Department of Psychology Queen's University Kingston Ontario Canada; ^12^ Department of Psychiatry Centre for Addiction and Mental Health, Campbell Family Mental Health Research Institute, Pharmacogenetic Research Clinic, University of Toronto Toronto Ontario Canada; ^13^ Department of Psychiatry, Faculty of Medicine University of Toronto Toronto Ontario Canada; ^14^ Department of Psychiatry, Krembil Research Centre University Health Network, University of Toronto Toronto Ontario Canada; ^15^ Department of Psychiatry, St. Michael's Hospital University of Toronto Toronto Ontario Canada; ^16^ Keenan Research Centre for Biomedical Science Li Ka Shing Knowledge Institute, St. Michael's Hospital Toronto Ontario Canada; ^17^ Department of Medical Biophysics, Faculty of Medicine University of Toronto Toronto Ontario Canada

**Keywords:** CAN‐BIND, emotional conflict, emotional Stroop, fMRI, ICC, test–retest reliability

## Abstract

Task‐based functional neuroimaging methods are increasingly being used to identify biomarkers of treatment response in psychiatric disorders. To facilitate meaningful interpretation of neural correlates of tasks and their potential changes with treatment over time, understanding the reliability of the blood‐oxygen‐level dependent (BOLD) signal of such tasks is essential. We assessed test–retest reliability of an emotional conflict task in healthy participants collected as part of the Canadian Biomarker Integration Network in Depression. Data for 36 participants, scanned at three time points (weeks 0, 2, and 8) were analyzed, and intra‐class correlation coefficients (ICC) were used to quantify reliability. We observed moderate reliability (median ICC values between 0.5 and 0.6), within occipital, parietal, and temporal regions, specifically for conditions of lower cognitive complexity, that is, face, congruent or incongruent trials. For these conditions, activation was also observed within frontal and sub‐cortical regions, however, their reliability was poor (median ICC < 0.2). Clinically relevant prognostic markers based on task‐based fMRI require high predictive accuracy at an individual level. For this to be achieved, reliability of BOLD responses needs to be high. We have shown that reliability of the BOLD response to an emotional conflict task in healthy individuals is moderate. Implications of these findings to further inform studies of treatment effects and biomarker discovery are discussed.

## 1 INTRODUCTION

Examining whether affective information interferes with the processing of cognitive information in individuals with major depressive disorder (MDD) is an area of investigation in mood disorder research, as emotional dysregulation may interfere with the effectiveness of cognitive control and interrupt cognitive activities (Schimmack & Derryberry, 2005). Studies of neural activation during emotion–cognition interference tasks in MDD may be useful to identify biomarkers. One such emotion–cognition interference task is the emotional conflict task (Egner, Etkin, Gale, & Hirsch, 2008; Etkin, Egner, Peraza, Kandel, & Hirsch, 2006), which includes an emotional Stroop‐like condition.

If the biomarker of interest is one that corresponds to treatment response, it is likely that longitudinal investigations will be required. Longitudinal studies assume that blood‐oxygen‐level dependent (BOLD) responses to a task are relatively stable within individuals and over time (Fournier, Chase, Almeida, & Phillips, 2014; Nord, Gray, Charpentier, Robinson, & Roiser, 2017). If that is the case, and if treatment is introduced between time‐points, changes in BOLD in response to a task could be interpreted as resulting from the intervention. Thus, to facilitate meaningful interpretation of the functional neural circuitry of cognitive and emotional processing over time, it is essential to first understand the test–retest reliability of the BOLD signal.

Reliability is generally accepted to be the consistency of a measure across repeated tests (Noble et al., 2017, p. 5415). Reliability can be assessed by various methods, including intraclass correlation coefficient (ICC), Pearson correlation, coefficient of variation, cluster overlap, or voxel counts (Aurich, Alves Filho, Marques da Silva, & Franco, 2015). In fMRI research, test–retest reliability has primarily been assessed using ICC (e.g., (Caceres, Hall, Zelaya, Williams, & Mehta, 2009; Elliott et al., 2019; Fournier et al., 2014). Studies assessing test–retest reliability of task‐based fMRI signals have yielded varied findings. For example, relatively consistent activations over time and between healthy participants have been reported for cognitive paradigms such as a probabilistic classification learning task (Aron, Gluck, & Poldrack, 2006) whereas other studies employing either a reward‐related guessing task (e.g., Chase et al., 2015), an emotion provocation task using neutral or fearful faces (e.g., Lipp, Murphy, Wise, & Caseras, 2014) or emotional face processing tasks (e.g., Nord et al., 2017) describe low test–retest reliability.

A recent meta‐analysis and independent analysis of task‐based fMRI data concludes that frequently used fMRI tasks do not show the test–retest reliability necessary for biomarker discovery (Elliott et al., 2019). They report an average ICC value of 0.397 across 90 studies in their meta‐analysis, which reflects poor reliability. Independent analyses of 11 commonly used tasks revealed ICCs of <0.3, again indicating rather poor test–retest reliability (Elliott et al., 2019). However, it also has to be noted that poor reliability may not necessarily render a measure unusable as a biomarker, as they may not automatically reflect changes in task performance but individual differences (Hedge, Powell, & Sumner, 2018).

Emotional conflict tasks activate fronto‐limbic circuitry, associating amygdala, cingulate, and prefrontal cortices with the generation, monitoring, and resolution of emotional conflict (Egner et al., 2008; Etkin et al., 2006). Reliability studies of tasks assessing emotional processing have shown poor reliability in the amygdala, ventral striatum, and cingulate cortices (e.g., Chase et al., 2015; Nord et al., 2017). If activation within this task is unstable with repeated testing in healthy participants, the analysis of such data for the purpose of defining biomarkers of treatment response may be problematic (Chase et al., 2015; Nord et al., 2017).

In this study, we assessed test–retest reliability of an emotional conflict task in healthy comparison participants collected within a Canadian Biomarker Integration Network in Depression (CAN‐BIND) protocol (Kennedy et al., 2019; Lam et al., 2016). The Canadian Biomarker Integration Network in Depression (CAN‐BIND‐1) Program aims to identify biomarkers of antidepressant treatment response in patients with major depressive disorder (MDD). The clinical protocol involves 8 weeks, open‐label treatment with the antidepressant escitalopram, which is followed by 8 weeks augmentation with the atypical antipsychotic aripiprazole in escitalopram non‐responders (see Kennedy et al., 2019; Lam et al., 2016). Participants were scanned three times (weeks 0, 2, and 8) and changes in activation within fronto‐limbic neural circuitry over time were assessed. We used intra‐class correlation coefficients (ICC) to quantify reliability. Specifically, we used ICC (3,1) because it treats systematic differences between repeat scans as fixed effects and is thus better suited to characterize biomarkers (Raemaekers et al., 2007). ICCs have been employed in previous fMRI studies assessing test–retest reliability of various experimental tasks, reporting fair reliability at best, with the majority of studies reporting ICC values between 0.33 and 0.66 (Bennett & Miller, 2010; Fournier et al., 2014). Low ICC values reflect low test–retest reliability of neural patterns over time. This is concerning because such changes cannot confidently be attributed to being caused by therapeutic effects. Misleading conclusions about the potential utility of using such tasks for the identification of biomarkers of treatment response may subsequently ensue (Fournier et al., 2014; Shadish, Cook, & Campbell, 2002).

## 2 METHODS

### 2.1 Participants

Fifty‐nine healthy control participants were recruited at academic healthcare centers across Canada as a subset of participants in the first Canadian Biomarker Integration Network in Depression study (CAN‐BIND‐1; (Kennedy et al., 2019; Lam et al., 2016)). Participants were aged between 18–60 years, had no psychiatric or unstable medical diagnoses and sufficient fluency in English to complete all study procedures. Demographic information for participants is listed in Table 1. Ethical approval was obtained from institutional ethics boards at each site, and informed consent was obtained from all participants. Useable neuroimaging data were available for 43 participants. Neuroimaging data were deemed unusable if it did not pass manual quality control (see MacQueen et al., 2019). Reasons for excluding scans were: excessive motion (*n* = 4), incomplete scan sequence (*n* = 1), severe ghosting or other data quality issues (*n* = 6). For *n* = 5, task‐based fMRI data was missing at one of the three time‐points or participants withdrew. In addition, for six participants the behavioral data were either not useable (e.g., reaction times were not recorded, no responses were made/recorded) (*n* = 1) (*n* = 3) or did not meet accuracy threshold (see below) (*n* = 2). This resulted in one site having only one participant contribute to the sample, so this participant was removed from further analysis. Hence, data were analyzed for 36 participants at three time points: weeks 0, 2, and 8. Mean time elapsed between week 0 and week 2 testing was 14.2 (±1.7) days; between week 2 and week 8 it was 42.5 (±3.6) days.

**Table 1:** Demographic information

Site	*N*	Age (mean ± *SD*)	Males:Females	Education(years)	Handedness left:right
University Health Network	12	31.6 (±10.3)	5:7	17.6 (±3.0)	1:11
McMaster University	4	42.5 (±5.8)	0:4	18.5 (±3.3)	0:4
Queens University	6	35.8 (±10.8)	1:5	18.2 (±3.0)	0:6
University of Calgary	14	30.3 (±8.8)	4:10	18.6 (±2.4)	2:12
Total	36	33.0 (±9.9)	10:26	18.2 (±2.7)	3:33

*Note*: There were no differences for age, *F*(3,32) = 2.0, *p* > .05 nor education, *F*(3,32) = 0.3, *p* > .05 between sites, nor for male/female ratios, chi‐square = 3.1, *p* > .05 nor handedness, chi‐square = 1.6, *p* > .05.

### 2.2 Design

The emotional conflict task (Egner et al., 2008; Etkin et al., 2006) assesses the cognitive cost that occurs when suppressing task irrelevant information to attend to task‐relevant information. The task comprises 148 black and white images (Ekman & Friesen, 1976) of either happy or fearful faces with the words “HAPPY” or “FEAR” superimposed on the images in bold red uppercase lettering. Stimuli were presented using E‐Prime software version 2 (https://pstnet.com/products/e-prime-legacy-versions/) and displayed on a projection screen. Participants viewed the screen via a mirror attached to a head‐coil. Stimuli were presented for 1 s and participants were instructed to identify the facial emotional expression via button press, as quickly and accurately as possible. Inter‐stimulus intervals, during which participants were instructed to look at a projected fixation cross, varied between 3 and 5 s and were jittered. Images were counterbalanced for equal numbers of congruent and incongruent presentations, in two consecutive “runs.” Participants completed the “runs” in the same order in each of their scan sessions to avoid between‐session variance associated with order. Each “run” comprised 74 stimuli and lasted 6 min 35 s. Prior to scanning, participants practiced the task outside of the scanner, to demonstrate understanding of task requirements.

Dependent variables were reaction time (RT) and accuracy. For the analysis of RT data error trials, post‐error trials (i.e., the trial following an error trial), and trials were RT exceeds two standard deviations above or below the trial type mean were not included. Commission error threshold was set at 25% per run, and the threshold of total allowable errors (combined omission and commission errors) was set at 30% per run. For the analysis of accuracy, trials were RT exceeded two standard deviations from the trial type mean and post‐error trials were included. For the neuroimaging analysis of all trials (e.g., all faces), all trials were included, regardless of RT or accuracy. Trial types analyzed included all face trials, which included error and post‐error trials, incongruent trials, congruent trials, incongruent minus congruent trials, fear minus happy face trials, fear minus happy word trials and iI minus cI trials which represent an incongruent trial being preceded by an incongruent trial (iI) minus an incongruent trial being preceded by congruent trial (cI). The main purpose of the “all faces” condition was to serve as control condition, rather than a main contrast of interest.

### 2.3 Data acquisition

Data were acquired at the University Health Network, Toronto, including Centre for Addiction and Mental Health; St. Joseph's Healthcare Hamilton; Providence Care Hospital, Kingston; the Mathison Centre for Mental Health Research & Education in the Hotchkiss Brain Institute, Calgary and Djavad Mowafaghian Centre for Brain Health, University of British Colombia, Vancouver. Three models of scanners were used across the clinical sites and included a Discovery MR750 3.0T (GE Healthcare, Little Chalfont, Buckinghamshire, UK), Signa HDxt 3.0T (GE Healthcare, Little Chalfont, Buckinghamshire, UK) and a MAGNETOM Trio (Siemens Healthcare, Erlangen, Germany). Each participant was scanned at the same site, using the same scanner across all three time points. Acquisition parameters are described in detail elsewhere ([MacQueen et al., 2019]) and summarized in Table 2. Since no participants from the University of British Colombia were included in the analyses (see Participants), this site is not included in Table 2.

**Table 2:** Scan parameters

CAN‐BIND site	Toronto Western/Toronto General Hospital	Centre for Addiction & Mental Health	McMaster University	University of Calgary	Queens University
**Scanner model**	GE 3.0T Signa HDxt		GE 3.0T Discovery MR750		Siemens 3.0T TrioTim
**Coil**	GE 8HRBRAIN	GE 8HRBRAIN	GE HNS head	GE HNS head	12‐channel head matrix coil
**T1 weighted structural sequence**					
TR (ms)	7.2	7.2–7.7	7.2–7.7	7.2–7.7	1,760–1900^a^
TE (ms)	2.7–2.9	2.7–2.9	2.7–2.9	2.7–2.9	2.2–2.7
TI (ms)	450	450	450	450	900–950^a^
Flip angle (degree)	15	15	15	15	15
Pixel bandwidth	244–260	244–260	122–260	244–260	199
Matrix dimension (pixels)	220 × 220–240 × 240	220 × 220–240 × 240	220 × 220–240 × 240	220 × 220–240 × 240	256 × 256
Voxel dimension (mm)	1 × 1 × 1	1 × 1 × 1	1 × 1 × 1	1 × 1 × 1	1 × 1 × 1
Number of slices	176	176–180	176–180	176–180	192
Acquisition time (min)	3:40	3:30	3:30	3:30	4:06
**fMRI—emotional conflict task**					
TR (ms)	2,000	2,000	2,000	2,000	2,000
TE (ms)	30	30	30	30	25
FOV	256	256	256	256	1,536 (mosaic)
Flip angle (degree)	75	75	75	75	75
Pixel bandwidth	7,812.50	7,812.50	7,812.50	7,812.50	2,232
Matrix dimension (pixels)	64 × 64	64 × 64	64 × 64	64 × 64	64 × 64
Voxel dimension (mm)	4 × 4 × 4	4 × 4 × 4	4 × 4 × 4	4 × 4 × 4	4 × 4 × 4
Number of volumes	202	202	202	202	202
Number of slices	34	40	36	36	40
Acquisition time (minutes)	6:44	6:44	6:44	6:44	6:44

Abbreviations: FOV, field of view; TE, echo time; TI, inversion time; TR, repetition time.

a The difference in parameters observed here is due to vendor specifications: In GE and Philips scanners, the TR represents an inner loop gradient echo TR, whereas in Siemens scanners the TR represents a magnetization‐prepared rapid gradient‐echo (MPRAGE) outer loop TR. (cf Potvin et al., 2019, p. 4.).

### 2.4 Data processing and analyses

#### 2.4.1 Data pre‐processing

Dicom images were converted to nifti, using mricron (http://people.cas.sc.edu/rorden/mricron/index.html). A sequence of fixed preprocessing and analysis steps were applied to the fMRI data as described by Churchill and colleagues (Churchill, Spring, Afshin‐Pour, Dong, & Strother, 2015) and included: (a) Estimation of the minimum‐displacement brain volume via Principal Component Analysis (PCA): We first determined the volume in which least head displacement occurred. A temporary copy of the data was then created, smoothed with a Gaussian kernel (FWHM = 6 mm), and its mean volume was removed. Then, PCA was performed, and the factors (multiplication of principal components [PCs] by their associated eigen‐values) were calculated. The median factor was calculated as the median of the factors for each time‐point. This is a robust measure of the center of the data. The mean distance of each volume from the estimated center point was then calculated, and the volume with the least distance was considered as the volume with the least head displacement. This was used as a reference for motion correction (step 3/iii) to minimize the average distance that each volume is displaced during alignment, as the accuracy of motion correction decreases with distance from the reference volume (Ardekani, Bachman, & Helpern, 2001). (b) Motion correction using rigid‐body motion correction (MOTCOR) as implemented in AFNI's 3dvolreg algorithm; (c) Identification of outliers using Censoring with outliers being either discarded or replaced with interpolated values from neighboring volumes: Basic censoring (CENSOR) was done by identifying significant outlier volumes in fMRI time series, which are discarded and replaced with interpolated values from neighboring volumes. This is done using the algorithm described in (Campbell, Grigg, Saverino, Churchill, & Grady, 2013) and was first validated for its impact on pipeline optimization by Churchill et al. (2015). It uses a robust sliding time‐window approach to identify outlier scans and replaces them with values interpolated from neighboring scans via cubic splines (stand‐alone software is available at: http://nitrc.org/projects/spikecor_fmri). (d) Slice‐timing correction (TIMECOR) with Fourier interpolation via AFNI's 3dTshift; (e) Spatial smoothing using AFNI's 3dBlurToFWHM to smooth fMRI images at FWHM = 6 mm in *x*,*y*,*z* directions allowing for different intrinsic reconstructed smoothing levels at each site: To match the spatial smoothing across MRI scanners at different sites, we used the 3dBlurToFWHM module in AFNI to smooth the fMRI images to the smoothness level of FWHM = 6 mm in three directions (*x*,*y*,*z*). Since the FWHM should reflect the spatial structure of the noise, we first regressed out the BOLD response modeled using the canonical hemodynamic response and then used the resultant residual image as the “blur master”. The “blur master” controls the process of smoothing on the original image. Blurring is applied to both the original image and the residual until the smoothness of the residual reaches the desired FWHM = 6 mm. Using a smoothing kernel with a FWHM ≈2× (in‐slice voxel width) has previously been shown to provide major improvements in prediction and reproducibility for group analysis of an fMRI motor task (S. Strother et al., 2004). (f) Obtaining a binary mask which excludes non‐brain voxels using AFNI's *3dAutomask* algorithm and applying the resultant mask to all EPI volumes; (g) Neuronal tissue masking: Neuronal tissue masking was performed by estimating a probabilistic mask to reduce the variance contribution of non‐neuronal tissues in the brain (e.g., macro‐vasculature, ventricles). This step uses the first part of the PHYCAA+ algorithm developed by Churchill and Strother (2013) to estimate task‐run and participant‐specific neural tissue masks (software available at http://nitrc.org/projects/phycaa_plus). (h) Calculation of nuisances regressors to be regressed‐out from the data concurrently via multiple linear regression: Temporal trends were modeled using a second‐order Legendre polynomial basis set, head motion effects on time‐series were modeled using participant motion parameter estimates (MPEs) obtained from MOTCOR, (Step 3). To obtain motion parameter regressors (MOTREG) per fMRI session, we performed PCA on the six MPE time‐courses, and used the largest‐variance principal components, which preserved 85% of the variance, as motion regressors. This allowed us to maximize the amount of head motion variance accounted for, while minimizing loss of power and collinearity effects due to unnecessary parameterization.

Preprocessed fMRI output files generated by these pre‐processing steps were in their original BOLD scan's brain space (*Native_processed‐fMRI*) but then transformed into normalized space (*sNORM_processed_fMRI*) to allow participants' results to be combined. For this, all scans were aligned to the MNI template (4 mm resolution) using FSL's FLIRT transformations. Subsequently, each participant's scans were transformed to MNI space. For more comprehensive discussions of these pipeline steps and their relative importance, see (Churchill, Raamana, Spring, & Strother, 2017; S. C. Strother, 2006).

#### 2.4.2 Analysis of behavioral data

Statistical analyses of the behavioral data were completed using SPSS 25 (IBM Corporation, 2017). Demographic data were analyzed using one‐way analyses of variances (ANOVA) for age and education and with chi‐square tests for sex and handedness. Behavioral data were analyzed using the Shapiro–Wilk test, to assess normality of the continuous variables, that is, RT and accuracy. Data were not normally distributed, and, therefore, nonparametric statistical analyses were conducted. Comparisons of paired samples were completed with the related‐samples Wilcoxon signed rank test. Friedman's test was used for within‐group analyses across all three time‐points. For within group comparisons, Wilcoxon signed rank tests or a sign test were conducted. To correct for multiple comparisons, Bonferroni corrections were applied, and adjusted *p*‐values are reported. Test–retest reliability of the behavioral data was assessed with intraclass correlation coefficients (ICC), using a two‐way random effects model for absolute agreement, and performed in SPSS v25. ICC is described in detail in Section 2.4.4. This model corresponds to the ICC (3,1) model as described by Fleiss & Shrout (see Hedge et al., 2018). Site effects were assessed; see supplementary information.

#### 2.4.3 Analysis of neuroimaging data

First‐level analyses for individual runs were completed using FSL FEAT v.6.00. Data were filtered with a high pass filter with a cut‐off of 100 Hz. Images were FILM prewhitened and a normal linear search with 3° of freedom (only translation) to the standard was applied to resample the images to the space of the 2 mm MNI 152 T1 standard brain.

Using Statistical Parametric Mapping 12 (SPM12; https://www.fil.ion.ucl.ac.uk/spm/, a higher‐level fixed‐effect model was constructed for each condition, separately for each time point, that is, weeks 0, 2, and 8, using the COPE images generated above. The model included regressors representing different trial types of the task: (a) all face trials, (b) congruent trials, (c) incongruent trials, (d) happy faces; (e) fear faces. Additionally, contrasts reflecting high and low conflict resolution trials, that is, incongruent (high conflict resolution) minus congruent (low conflict resolution) trials to measure the emotional Stroop condition, fear minus happy face trials, and iI minus cI trials were generated. It is thought that the comparison of “high conflict resolution > low conflict resolution” identifies regions associated with “conflict resolution” whereas the contrast “low conflict resolution > high conflict resolution” would identify regions implicated either in the “generation of conflict” or the “monitoring of conflict.” Second‐level analyses across participants (but within each time point, that is, weeks 0, 2, and 8) were conducted in SPM12, using random‐effects analyses with the above contrasts. For all conditions and contrasts tested, a cluster‐level threshold to control for multiple comparisons was set at *p* < .05—family‐wise‐error (FWE)‐corrected, with a cluster size of 10 or more voxels.

#### 2.4.4 Reliability analyses

Test–retest reliability of brain activation was assessed using intraclass correlation coefficients (ICC) (Shrout & Fleiss, 1979), a standard method to quantify the reliability of measurements between multiple test sessions (Bennett & Miller, 2010). ICC describe the stability of inter‐individual differences in brain activation over time, assessing within‐subject variance (σ within) relative to between‐subject variance (*σ* between):

$$\mathrm{ICC}\;\left(3,1\right)=\frac{\sigma_{\text{between}}^{2}-\sigma_{\text{within}}^{2}}{\sigma_{\text{between}}^{2}+\sigma_{\text{within}}^{2}}$$

Variance components were calculated by the individual contrast values separately for each trial type, and for each time point, that is, weeks 0, 2, and 8. Participants were treated as random effects and sessions (time‐points) were treated as fixed effects. ICCs can be interpreted as a ratio of variance (Bartko, 1966). ICCs approaching 1.0 suggest near‐perfect agreement between test and re‐test measurements, that is, relative neural activation is consistent across time‐points, whereas ICCs approaching 0 suggest no reliability. A negative ICC reflects a reliability of zero (Bartko, 1966), and can occur when the within‐group variance exceeds the between‐group variance (Lahey, Downey, & Saal, 1983). We assessed reliability using ICC (3,1), a measure of relative reliability as is appropriate for multi‐site fMRI data (Forsyth et al., 2014). ICC (3,1) measures the consistency between the repeated measurements, not the absolute agreement between them. ICCs were calculated for each voxel using the MATLAB‐based ICC toolbox (Caceres et al., 2009). Following Caceres et al., the median ICC for each cluster was considered as the primary reliability statistic of interest for that particular region. Median ICC was extracted for each significantly activated cluster, that is, based on the activity contrasts rather than ICC maps. Reliability was classified as “poor” (ICC < 0.4), “moderate to good reliability” (ICC = 0.4 to 0.75) or “excellent” (ICC > 0.75) (Nord et al., 2017).

We conducted whole‐brain analyses using the neuromorphometric atlas as implemented in SPM, to explore whether additional regions that may not have survived significance thresholds set in our analysis of contrasts showed higher reliability. The neuromorphometric template is based on maximum probability tissue labels derived from the MICCAI 2012 Grand Challenge and Workshop on Multi‐Atlas Labeling (https://masi.vuse.vanderbilt.edu/workshop2012/index.php/Challenge_Details) and available via SPM, provided by Neuromorphometrics, Inc. (http://neuromorphometrics.com/) under academic subscription. Exploring reliability across the whole‐brain is relevant when few anatomical or functional constraints have been established a priori (Noble et al., 2017). We therefore computed the median ICC for each region within the neuromorphometric atlas (number of regions = 136) for the contrasts described above.

## 3 RESULTS

### 3.1 Behavioral data

There were no significant changes in overall reaction time (RT) or overall accuracy across time, and neither RT nor accuracy changed over time for congruent or incongruent trials, all *p* > .05. A robust emotional Stroop effect was observed for RT and accuracy at each time point (see Table 3). Although there were no differences in RT between happy and fear faces at week 0, participants were significantly faster in responding to happy relative to fear faces at weeks 2 and 8 (see Table 4). Accuracy rates did not differ for happy or fear faces at any of the time points. Sex differences were not assessed due to small sub‐group numbers.

**Table 3:** Within‐group RT and accuracy on congruent and incongruent trials

	Congruent	Incongruent	Statistics
**RT**			
Week 0	671 ms	723 ms	*z* = 4.167, *p* < .0005*
Week 2	662 ms	722 ms	*z* = 5.833, *p* < .0005*
Week 8	670 ms	727 ms	*z* = 5.500, *p* < .0005*
**Accuracy**			
Week 0	0.98	0.95	*z* = −4.282, *p* < .0005*
Week 2	0.97	0.95	*z* = −2.437, *p* = .015
Week 8	0.98	0.95	*z* = −2.915, *p* = .004*

*Note*: Data are reported as median.

* Significant at *p* = .0125 (Bonferroni corrected).

**Table 4:** Within‐group RT on happy and fearful trials

	Happy	Fear	Statistics
**RT**			
Week 0	675 ms	709 ms	*z* = 1.500, *p* = .134
Week 2	679 ms	715 ms	*z* = 3.167, *p* = .002*
Week 8	694 ms	709 ms	*z* = 2.500, *p* = .012*
**Accuracy**			
Week 0	0.97	0.96	*z* = −.697, *p* = .486
Week 2	0.96	0.95	*z* = −.225, *p* = .822
Week 8	0.97	0.96	*z* = −.884, *p* = .377

*Note*: Data are reported as median.

* Significant at *p* = .0125 (Bonferroni corrected).

The ICCs of the behavioral measures are listed in Table 5. None of these demonstrated excellent reliability (i.e., ICCs of 0.8 or above), but several of these measures demonstrated good reliability, with ICCs being between 0.5 and 0.6.

**Table 5:** Test–retest reliability of behavioral data

Measure	ICC	95% confidence interval
**Reaction time**		
Congruent trials	0.681	0.522–0.808
Incongruent trials	0.642	0.472–0.781
Happy faces	0.657	0.491–0.791
Fear faces	0.666	0.503–0.798
**Accuracy**		
Congruent trials	0.460	0.258–0.650
Incongruent trials	0.577	0.391–0.736
Fear faces	0.622	0.446–0.768
Happy faces	0.429	0.223–0.626
**Contrasts (reaction times)**		
Incongruent versus congruent	0.357	−0.106‐0.648
Fear versus happy	0.663	0.417–0.816
iI versus cI	0.437	0.010–0.696
**Contrasts (accuracy)**		
Incongruent versus congruent	0.583	0.280–0.772
Fear versus happy faces	0.454	0.046–0.705
iI versus cI	0.028	−0.708‐0.475

*Note*: Typical interpretation of ICC values are: 0.8 = excellent reliability; 0.6 = good reliability; 0.4 = moderate reliability (Cicchetti & Sparrow, 1981; Fleiss, 1981; Landis & Koch, 1977).

Abbreviation: ICC, intraclass correlation coefficient.

### 3.2 Neuroimaging data

#### 3.2.1 All faces trials

**Figure 1:**
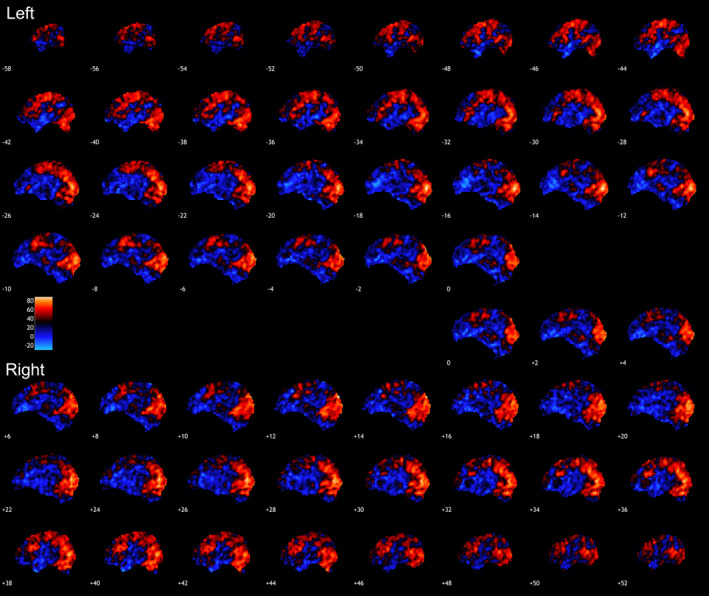
All faces condition. Presented are voxel‐based ICC maps for the condition tested displayed in the sagittal planes. Warmer colors indicate higher ICC. Highest ICCs were observed in visual regions, including lingual gyrus; also, in precuneus and middle temporal regions. ICC, intraclass correlation coefficient

The voxel‐wise ICC estimates across the three time‐points were moderate, at best, for regions showing significant BOLD activation (*p*_FWE‐corrected_ = .05) for the “*all faces trials*” contrast (see Table 5 and Figure 1). We observed moderate reliability (median ICC = 0.5) within visual regions, including the right lingual gyrus and the right precuneus as well as the left middle temporal gyrus. The right inferior frontal gyrus and superior temporal gyrus, in addition to the left parahippocampal gyrus and the left insula median had modest ICC values of 0.4. The right parietal regions (postcentral gyrus, precuneus), bilateral temporal regions (right superior temporal gyrus; left fusiform), bilateral frontal regions (left medial frontal gyrus, right inferior and middle frontal gyri) and the left posterior cingulate, insula, and claustrum and the right thalamus all had poor ICC values (ICC ≤ 0.4).

#### 3.2.2 Congruent face trials

**Figure 2:**
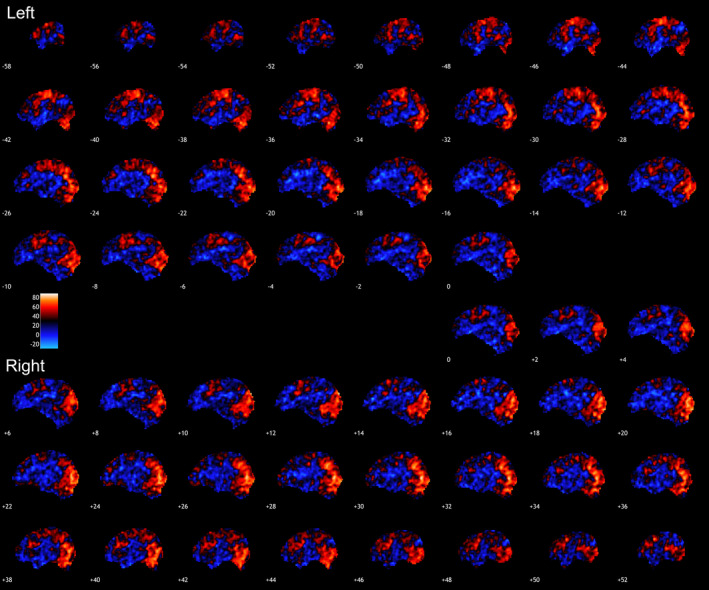
Congruent trials condition. Presented are voxel‐based ICC for the condition tested displayed in the sagittal planes. Warmer colors indicate higher ICC. Highest ICCs were observed in fusiform and the middle temporal gyri, as well as the right precuneus and the left post‐central gyrus. ICC, intraclass correlation coefficient

Significant activation (*p*_FWE‐corrected_ = .05) was observed in the fusiform gyrus and the middle temporal gyrus, as well as the right precuneus and the left post‐central gyrus for *congruent faces* (see Table 5 and Figure 2). Neural activation within these regions showed moderate reliability, with median ICCs = 0.5. Additionally, significant activation (*p*_FWE‐corrected_ = .05) was observed within frontal and sub‐cortical regions such as the right precentral gyrus and the left cingulate gyrus and the left insula. In these areas, ICC values were 0.4. Poor reliability was observed for significant activation within the right thalamus, pyramis, insula, the right postcentral gyrus and the right superior frontal and right superior temporal gyri, as well as the left insula and the left parahippocampal gyrus.

#### 3.2.3 Incongruent face trials

**Figure 3:**
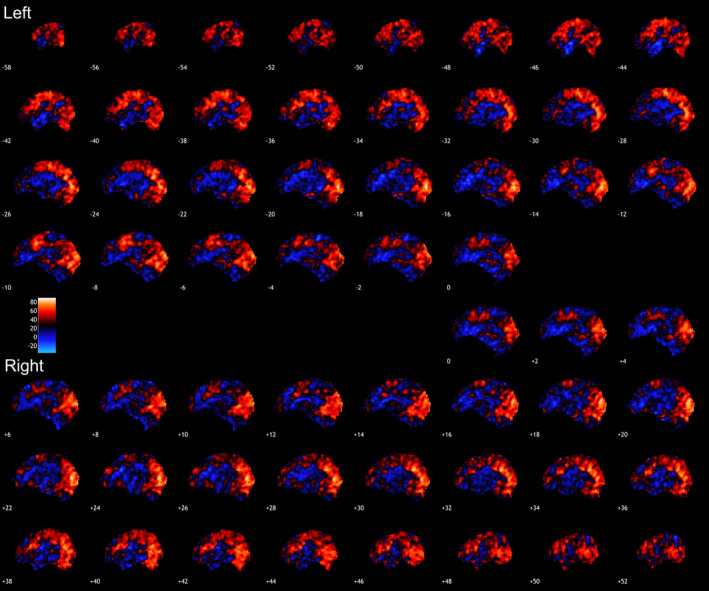
Incongruent trials condition. Presented are voxel‐based ICC for the condition tested displayed in the sagittal planes. Warmer colors indicate higher ICC. Highest ICCs were observed in precuneus and superior parietal regions. ICC, intraclass correlation coefficient

The left middle temporal gyrus, right precuneus and right superior parietal lobule had significant activation (*p*_FWE‐corrected_ = .05) for *incongruent faces* (Table 5, Figure 3). Reliability was moderate in these regions, with median ICCs = 0.5. Significant activation was also apparent in the right inferior frontal gyrus, the superior temporal gyrus and within the thalamus, but ICC values for these regions were modest at 0.4. Poor reliability was observed within the left insula and the right postcentral gyrus, median ICC = 0.3.

#### 3.2.4 Fear minus happy face trials

The voxel‐wise ICC estimates for more cognitively demanding comparisons, specifically for the *fear minus happy* contrasts for both trials with the word fear minus the word happy and trials with a fear face minus happy face conditions, were very poor (median ICC = 0.1; Table 5).

For the *fear word minus happy word* contrast, significant activation (*p*_FWE‐corrected_ = .05) was observed within the right supramarginal gyrus and the right inferior parietal lobule.

For the *fear face minus happy face* contrasts, significant activation (*p*_FWE‐corrected_ = .05) was observed within right superior and middle temporal gyri.

#### 3.2.5 Incongruent minus congruent trials

For the *incongruent minus congruent* contrast, significant activation (*p*_FWE‐corrected_ = .05) was observed for the right inferior parietal lobule, the left precuneus and the left cingulate, as well as within the left medial and precentral gyri and the inferior frontal gyrus, bilaterally. Median ICC values within these regions were ≤0.1 (Table 5).

#### 3.2.6 iI minus cI trials

For the *iI* (an incongruent trial preceded by an incongruent trial) *minus cI* (incongruent trial preceded by a congruent trial) contrast, no significant results were obtained when using FWE‐corrected threshold. To facilitate ICC analyses, reported here are the results from an uncorrected threshold, *p*_uncorrected_ = .001, observed within bilateral cingulate gyri. However, median ICC values were poor (Table 5).

### 3.3 Exploratory analyses: Whole‐brain analyses using neuro‐morphometric atlas

The voxel‐wise ICC estimates across the three time‐points for the “*all faces*” contrasts were most reliable within visual regions, including the calcarine cortex, the lingual gyri, cuneus, and the inferior occipital gyri, all bilaterally, all with ICC = 0.6, and within the occipital fusiform gyri (bilaterally; left ICC = 0.5; right ICC = 0.5). For *congruent* trials only, the pattern was similar and higher ICC values were observed within visual regions, including bilateral cuneus (left ICC = 0.4; right ICC = 0.5), bilateral occipital fusiform gyri (ICC = 0.5), bilateral calcarine cortex (ICC = 0.5), bilateral lingual gyri (ICC = 0.5), bilateral inferior occipital gyri (left ICC = 0.4; right ICC = 0.5), bilateral superior occipital gyrus (left ICC = 0.4; right ICC = 0.5). The pattern was similar for *incongruent* trials; ICC values between 0.5 and 0.6 were observed for the cuneus (bilaterally, ICC = 0.6), bilateral occipital fusiform gyri (ICC = 0.5), bilateral calcarine cortex (left ICC = 0.5, right ICC = 0.6), bilateral lingual gyri (ICC = 0.5), bilateral inferior occipital gyri (ICC = 0.5) and bilateral superior occipital gyrus (ICC = 0.5).

The voxel‐wise ICC estimates across all three time‐points for all cognitively more demanding contrasts were poor, ICC values ≤0.2.

## 4 DISCUSSION

The aim of this study was to examine the reliability of the BOLD signal for an emotional conflict task as a prerequisite to assessing the task's suitability to establish biomarkers of treatment response in clinical populations. Comparing across three time‐points, weeks 0, 2 and 8, we observed moderate reliability (median ICC values between 0.5 and 0.6) within occipital, parietal and temporal regions, specifically for conditions of lower cognitive complexity, such as all faces, and congruent or incongruent trials relative to baseline. Activation was also observed within frontal and sub‐cortical regions for the same conditions, but the median ICC values were poor. We did not observe “good” or “excellent” reliability for any regions. Median ICC values of 0.5 and 0.6 were also calculated for the lingual gyri, cuneus and occipital fusiform gyri for less cognitively demanding conditions, whereas poor reliability was observed for contrasts demanding more cognitive processing when using the neuromorphometrics template in exploratory analyses.

Our findings are consistent with previous reports (Chase et al., 2015; Fournier et al., 2014; Lipp et al., 2014; Nord et al., 2017) as well as a current meta‐analysis (Elliott et al., 2019). Elliott et al. (2019) reported an average ICC of 0.397 for unthresholded ICC estimates across a variety of task‐based fMRI studies. Additionally, Nord et al. (2017), reported very low ICC values for three emotional processing tasks in regions activated by the tasks such as the ACC and the amygdala. Reliability was better in control regions such as visual processing regions, with ICC values of ≥0.7 (Nord et al., 2017). The observed significant activation and moderate reliability of regions such as the lingual gyrus, the cuneus or occipital fusiform gyri in our task is therefore unsurprising, given their involvement in visual processing. The lingual gyri have been implicated in the processing of facial expressions of emotions (Kitada, Johnsrude, Kochiyama, & Lederman, 2010) but also letters, both forming part of the stimuli used here. The lingual gyrus may also have a role in the analysis of logical conditions (Mechelli, Humphreys, Mayall, Olson, & Price, 2000). The nature of trials in our task, congruent but also incongruent, may have contributed to activation of this region.

The cuneus is a primary visual area involved in response inhibition by contributing to motor responses rather than error monitoring (Booth et al., 2005; Haldane, Cunningham, Androutsos, & Frangou, 2008; Matthews, Simmons, Arce, & Paulus, 2005). The Stroop‐like nature of this task required careful selection of an appropriate response that may have facilitated the activation in the cuneus. The occipital fusiform gyrus (FFG) is involved primarily in higher functions of vision, for example, differentiating between different categories of objects, perception and recognition of faces (Kanwisher & Yovel, 2006; Ma & Han, 2012), but its left lateral portion also contributes to the recognition of visual words and reading (Dehaene & Cohen, 2011; McCandliss, Cohen, & Dehaene, 2003). Thus, the activation of these areas is again not surprising since the stimuli used in this task involved both of these features. Parietal regions, such as the precuneus, the post‐central gyrus and the superior parietal lobule also showed moderate reliability, primarily in response to the all face condition or the congruent or incongruent conditions. The precuneus has a role in visuo‐spatial imagery, episodic memory retrieval and self‐processing operations (Cavanna & Trimble, 2006) and has been implicated in this task during the contrasting of congruent and incongruent conditions (Fournier et al., 2017). Fournier and colleagues explained that activation within this area is linked to switching between easier, congruent trials – which may reflect default processing (given the precuneus's role as a node of the default mode network) – to more complex, incongruent trials (Fournier et al., 2017).

The superior parietal lobule is involved with processing visual information as it relates to spatial orientation (Corbetta, Kincade, Ollinger, McAvoy, & Shulman, 2000). Temporal regions such as the middle temporal gyrus showed significant activation in response to all faces but also congruent and incongruent conditions. This region is involved in the recognition of known faces but has also been linked to accessing word meaning while reading (Acheson & Hagoort, 2013). Again, given that the stimuli used in this study comprise both faces and words, it is not surprising that activation in these areas was observed.

We detected activation in brain areas expected to be implicated in this task, for example, inferior and orbital prefrontal cortical (PFC) regions such as Brodmann area (BA) 44 and BA47, dorso‐lateral and anterior PFC regions such as BA46 and BA10, in addition to subcortical structures, such as the cingulate gyri, the insula and the parahippocampus/amygdala (see Table 6). Within these regions, and in particular in response to cognitively more complex contrasts, such as “*iI* < *cI*” or “*incongruent* < *congruent*” trials, reliability was poor (median ICC ≤ 0.1). Activation in BA44 has previously been linked to selective response suppression in response‐inhibition tasks, such as a go/no‐go task (Forstmann, van den Wildenberg, & Ridderinkhof, 2008) as well as to hand‐movements (Rizzolatti, Fogassi, & Gallese, 2002). The dorso‐lateral and anterior prefrontal regions are implicated in task‐aspects such as sustained attention and executive processing. Both insula and cingulate subserve the task employed here. Egner and colleagues reported activation of the cingulate regions for the iI minus cI contrast, which is also referred to as conflict monitoring. Here we also observed significant activation in both the dorsal anterior and posterior cingulate in response to “*iI* versus *cI*” trials, that is, high‐conflict versus low‐conflict trials (Egner et al., 2008). However, ICC values for these regions were poor.

**Table 6:** Neuroimaging data on all conditions and contrasts conducted

*K*	FWE corrected *p*	Region				*t*	*x*	*y*	*z*	Median ICC
**All faces**										
32,666	.000	Right	Occipital lobe	Lingual gyrus	BA18	17.16	14	−82	−12	0.51
3,450	.000	Right	Frontal lobe	Inferior frontal gyrus	BA46	10.88	46	34	16	0.37
588	.000	Right	Temporal lobe	Superior temporal gyrus	BA22	8.9	60	−42	12	0.39
80	.000	Right	Parietal lobe	Postcentral gyrus	BA43	8.24	58	−14	16	0.19
189	.000	Right	Parietal lobe	Precuneus	BA7	8.16	14	−70	40	0.5
*58*	*.005*	*Left*	*Limbic lobe*	*Parahippocampal gyrus/Amygdala*		*8*	*−20*	*−6*	*−16*	*0.35*
15	.001	Right	Temporal lobe	Superior temporal gyrus	BA13	7.4	46	−20	8	0.21
*28*	*.002*	*Left*	*Limbic lobe*	*Posterior cingulate*	*BA23*	*7.14*	*−4*	*−32*	*24*	*0.1*
12,694	.000	Left	Temporal lobe	Fusiform gyrus	BA37	14.61	−38	−52	−22	0.29
6,043	.000	Left	Frontal lobe	Medial frontal gyrus	BA6	13.31	−2	−2	56	0.34
1995	.000	Right		Thalamus		12.02	22	−30	−4	0.13
2,309	.000	Right	Frontal lobe	Inferior frontal gyrus	BA47	11.27	34	26	0	0.27
450	.000	Right	Parietal lobe	Precuneus	BA7	11.02	30	−52	44	0.34
487	.000	Left		Insula	BA13	10.46	−34	18	4	0.39
57	.000	Left		Insula	BA13	9.62	−50	−40	20	0.08
333	.000	Right	Temporal lobe	Superior temporal gyrus	BA22	8.46	46	−30	−4	0.23
26	.000	Left	Temporal lobe	Middle temporal gyrus	BA22	8.35	−50	−44	4	0.46
84	.000	Right	Frontal lobe	Middle frontal gyrus	BA10	8.17	30	42	20	0.14
39	.000	Right	Parietal lobe	Postcentral gyrus	BA43	7.34	58	−14	18	0.18
**Congruent**										
13,494	.000	Left	Temporal lobe	Fusiform gyrus	BA37	15.89	−38	−52	−22	0.51
434	.000	Right		Thalamus		12.8	22	−30	−4	0.18
455	.000	Right	Parietal lobe	Precuneus	BA7	12.44	30	−50	44	0.48
1807	.000	Left	Limbic lobe	Cingulate gyrus	BA24	11.8	−2	−4	50	0.36
722	.000	Left	Limbic lobe	Parahippocampal gyrus	BA27	11.64	−22	−30	−8	0.22
4,116	.000	Left	Parietal lobe	Postcentral gyrus	BA3	10.48	−54	−30	40	0.45
71	.000	Left		Insula	BA13	10.11	−50	−40	20	0.17
176	.000	Left		Claustrum		9.62	−32	18	4	0.31
58	.000	Right	Cerebellum	Pyramis		9.41	8	−74	−40	0.16
349	.000	Right	Frontal lobe	Precentral gyrus	BA6	8.74	46	−6	54	0.41
58	.000	Right	Parietal lobe	Postcentral gyrus	BA43	8.67	58	−14	16	0.29
36	.000	Left		Insula	BA13	8.63	−36	26	20	0.36
78	.000	Right		Insula	BA13	7.85	36	14	0	0.31
30	.000	Left	Temporal lobe	Middle temporal gyrus	BA22	7.74	−50	−44	4	0.45
124	.000	Right	Temporal lobe	Superior temporal gyrus	BA22	7.52	48	−26	−2	0.22
**Incongruent**										
15,383	.000	Left	Temporal lobe	Fusiform gyrus	BA37	17.77	−38	−50	−22	0
2,834	.000	Left	Limbic lobe	Cingulate gyrus	BA24	14.18	−2	−2	52	
7,682	.000	Right		Thalamus		13.01	20	−30	−4	0.41
678	.000	Right	Parietal lobe	Superior parietal lobule	BA7	12.39	32	−54	48	0.48
2,630	.000	Right	Frontal lobe	Inferior frontal gyrus	BA46	9.71	48	30	14	0.35
165	.000	Left	Temporal lobe	Middle temporal gyrus	BA22	9.65	−50	−44	4	0.47
87	.000	Right	Parietal lobe	Postcentral gyrus	BA43	9.41	56	−14	20	0.24
349	.000	Right	Temporal lobe	Superior temporal gyrus	BA22	8.88	62	−38	12	0.37
62	.000	Left		Insula	BA13	8.8	−50	−40	20	0.27
74	.000	Right	Parietal lobe	Postcentral gyrus	BA3	8.13	54	−20	38	0.33
95	.000	Right	Parietal lobe	Precuneus	BA7	7.84	22	−70	34	0.5
**Incongruent minus congruent**										
276	.001	Right	Parietal lobe	Inferior parietal lobule	BA40	5.5	40	−56	46	0.02
*158*	*.020*	*Left*	*Frontal lobe*	*Medial frontal gyrus*	*BA6*	*5.23*	*−12*	*−14*	*70*	*0.02*
*148*	*.026*	*Left*	*Parietal lobe*	*Precuneus*	*BA7*	*5.14*	*−22*	*−64*	*42*	*0.06*
1,546	.000	Right	Frontal lobe	Inferior frontal gyrus	BA44	5.13	58	18	16	0.12
365	.000	Left	Limbic lobe	Cingulate gyrus	BA32	5.06	−10	14	38	0
*150*	*.025*	*Left*	*Frontal lobe*	*Inferior frontal gyrus*	*BA47*	*5.06*	*−40*	*30*	*−2*	*−0.07*
455	.000	Left	Frontal lobe	Precentral gyrus	BA6	4.96	−42	−8	42	0.11
**Word: Fear minus happy**										
*154*	*.014*	*Right*	*Parietal lobe*	*Supramarginal gyrus*	*BA40*	*4.8*	*64*	*−48*	*30*	*−0.06*
		*Right*	*Parietal lobe*	*Inferior parietal lobule*	*BA40*		*62*	*−40*	*42*	*0*
**Face: Fear minus happy**										
*162*	*.011*	*Right*	*Temporal lobe*	*Superior temporal gyrus*	*BA22*	*5.47*	*48*	*−38*	*2*	*0.13*
		*Right*	*Temporal lobe*	*Middle temporal gyrus*	*BA21*	*3.91*	*52*	*−50*	*2*	*0*

*K*	Peak *p* (unc)	Region				*t*	*x*	*y*	*z*	Median ICC
**iI minus cI**										
8	.000	Right	Limbic lobe	Cingulate gyrus	BA31	4.01	24	−28	36	0
3	.000	Left	Limbic lobe	Cingulate gyrus	BA32	3.75	−16	8	42	0
4	.001	Left	Temporal lobe	Hippocampus		3.53	−30	−28	6	0
2	.001	Left	Limbic lobe	Anterior cingulate	BA32	3.39	−20	32	16	0

*Note: Italics*: FWE‐corrected *p*‐value = .05; for iI minus cI results from an uncorrected threshold, *p* uncorrected = .001 are shown.

Abbreviations: BA, Brodmann area; cI, congruent trial preceded by an incongruent trial; FWE, family‐wise‐error; ICC, intraclass correlation coefficient; iI, incongruent trial preceded by an incongruent trial; *K*, cluster extent; unc, uncorrected.

Insula activation was observed in response to both incongruent and congruent trials, as well as in the all faces versus baseline condition. The insula is involved in several cognitive as well as emotional processes, interoception (Critchley, Wiens, Rotshtein, Öhman, & Dolan, 2004), and social emotions (Quarto et al., 2016; Sanfey, Rilling, Aronson, Nystrom, & Cohen, 2003). It is also an important node in the salience network, in relation to response selection and selective attention. Insula activation was previously reported in response to incongruent minus congruent trials in an MDD group (Fournier et al., 2017).

The lack of convincing reliability observed here in a substantial number of conditions and contrasts has significant implications for studies using task‐based fMRI to identify biomarkers of treatment response for any psychiatric disorder, not just depression. For the most part, we observed activation in regions that are both understandable given the nature of the task and consistent with previous reports using this task. To that extent, the task met expectations in healthy comparison participants. Nonetheless, reliable activation of key regions across repeated testing in healthy participants was not apparent. Our results are in line with the conclusions of a recent meta‐analyses and subsequent confirmatory findings of poor test–retest reliability in a variety of fMRI‐based tasks (Elliott et al., 2019). This suggests that the suitability of such a task for uncovering biomarkers of treatment response in any patient population using repeated measures is questionable, as any associations with treatment would have to be distinguishable from fluctuations in activation that appear to be inherent to the task.

These results are consistent with test–retest reliability studies of other task‐based and resting‐state fMRI, which have reported reliability in the poor to good range (Chase et al., 2015; Fournier et al., 2014; Lipp et al., 2014; Noble et al., 2017; Nord et al., 2017; Plichta et al., 2012; Shah, Cramer, Ferguson, Birn, & Anderson, 2016; Shehzad et al., 2009; Shou et al., 2013). In our cohort, reliability was better (i.e., moderate) in cortical regions, but typically poor in sub‐cortical structures, corresponding to previous reports (Fournier et al., 2014). Non‐cortical regions may overall be less reliable (Shah et al., 2016) because of the smaller sizes of sub‐cortical structures (Noble, Spann, et al., 2017). Furthermore, it has been reported that the magnitude of ICC is influenced by the complexity of the functional contrasts investigated (e.g., Brown et al., 2011). We observed that reliability was higher for conditions that were less cognitively demanding (e.g., all faces versus baseline, congruent or incongruent trials relative to baseline) than for contrasts that were related to higher cognitive demand, for example, conflict monitoring or the emotional Stroop effect. Trials of less cognitive complexity are thought to retain more of the BOLD signal relative to higher cognitive complexity contrasts for which potentially larger subtractions of neural activity result in less BOLD signal, subsequently reducing ICC (Brown et al., 2011). Thus, considering a trade‐off between the complexity of the model, or contrast, and its interpretability is important when assessing test–retest reliability, especially when an intervention is introduced between scans. Direct comparison of active contrasts (e.g., incongruent minus congruent) showed poor reliability also in other neuroimaging studies (e.g., [Infantolino, Luking, Sauder, Curtin, & Hajcak, 2018]); however, main effects, or conditions, such as congruent or incongruent showed comparatively better reliability. Furthermore, observed differences in reliability may also be related to the nature of the task and more so to the similarity of its trials. For example, the fear versus happy face contrasts may show similar variance or correlations which may subsequently appear less reliable, see Hedge et al. (2018), who states that measures may be less reliable when highly correlated or of similar variance. Indeed, assessments of ICCs for the behavioral equivalent of the neuroimaging contrasts (e.g., accuracy for fear vs. happy; incongruent vs. congruent) showed moderate reliability (0.45 and 0.58, respectively). This is comparable to the ICCs of the behavioral data for the overall conditions. However, for reaction time, ICC for the incongruent versus congruent contrast was reduced relative to the overall conditions, further supporting findings of Infantolino et al. (2018) and Hedge et al. (2018), that in addition to examining the reliability of neural measures, the behavioral measures should be assessed as well. Additionally, the fact that some conditions (e.g., all faces, congruent or incongruent) had a larger number of trials than other contrasts, such as conflict monitoring, needs to be considered; poor reliability in those trials may, at least in part, relate to low statistical power (Brown et al., 2011).

Whole‐brain neuromorphometric analyses suggested that the most reliable regions (e.g., visual/occipital regions) may not be regions related to task‐relevant activation (e.g., cingulate cortex). Noble and colleagues, using resting state fMRI, reported similar observations, stating that the most reliable edges are not necessarily the most informative ones, and vice versa (Noble et al., 2017). Likewise, Plichta et al. (2012) reported that in response to three different tasks (a reward task, a faces task and an n‐back task) the voxels responding most strongly were also not necessarily the ones showing the most reliable pattern of activation, and that voxels showing high ICC values were observed in regions not necessarily engaged by the task. Therefore, it may be possible that meaningful information unique to each individual could be captured by data with relatively low test–retest reliability. This may, however, hinder the development of fMRI predictive biomarkers of treatment response (Noble et al., 2017).

The strengths of this study include the three time‐points for a sample size (*n* = 36) that represents at least a comparable number of participants to those examined in previous studies. Noble and colleagues (2017) scanned 12 participants on four occasions, Nord et al. (2017) scanned 29 participants twice, Lipp et al. (2014) scanned 15 participants twice and Chase et al. (2015) scanned 37 participants twice. The duration between test–retest sessions has been argued to influence reliability. For example, Fournier et al. (2014) argue that it is possible that their 6‐month interval between scans reduced reliability. Here, we assessed reliability across three time points with the longest duration between scans being 6 weeks (week 2 to week 8) and this may be partly why the reliability reported here was marginally better. However, our assessment of reliability from week 0 to week 2 did not substantially improve reliability (see supplementary information). This is consistent with conclusions from Elliott et al. (2019) who reported that the test–retest interval had little impact on reliability estimates. Furthermore, we collected data from multiple sites using different scanners, and employed considerable efforts to maintain high image quality. This may be of potential benefit, since it might improve and demonstrate generalizability of our results. Post‐hoc analyses of site effects (see supplementary information) showed that site by time interactions were not significant.

### 4.1 Limitations

The methods employed to assess reliability need to be taken into consideration when interpreting our findings in the wider context. For one, results are reported for the reliability of clusters obtained from univariate GLM statistics. It has previously been reported that not only modeling approaches chosen for analyses (Fournier et al., 2014), but also the selection of preprocessing steps (Churchill et al., 2015) affect signal detection and subsequently test–retest reliability. Studies, primarily using resting‐state fMRI data, have shown that pre‐processing parameters, such as censoring based on outliers within functional time‐series, impact reliability estimates of connectivity measures (e.g., Aurich et al., 2015). Evaluating the effect of pre‐processing pipelines on reliability measures of task‐based fMRI data could thus be of future interest.

We employed an FWE‐corrected threshold of *p* < .05, which has previously been regarded as too lenient (Eklund, Nichols, & Knutson, 2016). Most clusters, however, were significant at *p* < .001, as evident in Table 6. Multi‐variate assessments of test–retest reliability have previously also been shown to improve reliability measures over univariate methods (Noble et al., 2017) and should thus be explored further in future.

Secondly, the measure employed here to assess test–retest, ICC, is a statistical estimate of reliability rather than a direct marker of test–retest stability. It can be affected by factors other than the underlying stability of the BOLD signal (Fournier et al., 2014). Furthermore, we have not analyzed the breakdown of the ICC variance and may therefore not completely be able to rule out additional effects of time on ICC measures.

In addition, homogenous samples, such as our group of healthy adult participants, may have reduced ICC estimates (Bennett & Miller, 2010), because of the way that ICCs are calculated. Assessing reliability in patient populations might provide additional tests of the utility of fMRI biomarkers in treatment response research, but as the CAN‐BIND study included an intervention for all patient participants, it was not possible to assess this in CAN‐BIND.

We performed whole‐brain approaches whereas previous studies assessing test–retest reliability mostly used region of interest analysis (e.g., Chase et al., 2015; Nord et al., 2017). Restraining our analyses to a priori defined regions might have improved ICC but our exploratory analysis using the neuromorphometrics template assessed amygdala and cingulate regions and showed poor reliability, comparable to previous observations (e.g., Nord et al., 2017).

## 5 CONCLUSION

In this study, the reliability of the BOLD signal in regions subserving an emotional conflict task was poor to moderate, despite behavioral and activation measures suggesting that the task performed as expected at all three time points. These results are consistent with other reports. Clinically relevant prognostic markers based on task‐based fMRI would require high predictive accuracy at an individual level and for this to be achieved, BOLD responses need to be highly reliable. Typical analyses of task‐based fMRI of cognitive‐emotional processes therefore appear to lack the reliability required to uncover biomarkers of treatment response in longitudinal clinical studies. However, it should also be considered that low reliability of task‐based fMRI markers may not necessarily mean their potential in biomarker discovery is lost, but that the reasons for this lack of consistency would need to be evaluated appropriately (Hedge et al., 2018). Novel analytic methods may be required to determine whether these tasks have utility as predictive tasks in clinical trials.

## DISCLOSURES AND CONFLICT OF INTEREST

Drs. *Hassel*, *Sharma*, *Davis*, *Arnott*, *Hall*, *Müller*, and *Rotzinger* have no conflict of interest to declare. Ms. *Alders*, Ms. *Harris*, and Ms. *Zamyadi* have conflict of interest to declare. Dr. *Frey* has received research funding from Pfizer*. Dr. Lam* has received honoraria for ad hoc speaking or advising/consulting, or received research funds, from: Akili, Allergan, Asia‐Pacific Economic Cooperation, BC Leading Edge Foundation, Canadian Institutes of Health Research, Canadian Network for Mood and Anxiety Treatments, Canadian Psychiatric Association, CME Institute, Hansoh, Healthy Minds Canada, Janssen, Lundbeck, Lundbeck Institute, Medscape, Mind.Me, MITACS, Ontario Brain Institute, Otsuka, Pfizer, St. Jude Medical, University Health Network Foundation, and VGH‐UBCH Foundation. Dr. *Milev* has received consulting and speaking honoraria from Allergan, Janssen, Lundbeck, Otsuka, Pfizer and Sunovion, and research grants from CAN‐BIND, CIHR, Janssen, Lallemand, Lundbeck, OBI, OMHF, and Pfizer. Dr. *Kennedy* has received research funding or honoraria from the following sources: Abbott, Alkermes, Allergan, BMS, Brain Canada, Canadian Institutes for Health Research, Janssen, Lundbeck, Lundbeck Institute, Ontario Brain Institute, Ontario Research Fund, Otsuka, Pfizer, Servier, Sunovion and Xian‐Janssen. Dr. *MacQueen* reports consultancy/speaker fees from Lundbeck, Pfizer, Johnson & Johnson and Janssen, outside the submitted work. Dr. *Strother* receives funding from the OBI and CIHR (MOP137097) for neuroimaging analysis in CAN‐BIND and he is the Chief Scientific Officer of ADMdx, Inc., a neuroimaging consulting company.

## Supporting information


**Appendix S1**: Supporting Information

## Data Availability

Data subject to third party restrictions. The data that support the findings of this study are available from the Ontario Brain Institute at a later date. Restrictions apply to the availability of these data, which were used under license for this study. Data will be made available with the permission of OBI at a later date.
