# Supplementary material for: Reliability of a functional magnetic resonance imaging task of emotional conflict in healthy participants
Source: Hum Brain Mapp. 2019 Dec 3;41(6):1400–15. doi: 10.1002/hbm.24883 (PMC7267954; doi:10.1002/hbm.24883)
Supplement: Supplementary file 1 — Appendix S1: Supporting Information [file HBM-41-1400-s001.docx]

**Supplementary Materials**

*Post-hoc Analysis of Site Differences*

To assess for the effects of site on demographic and behavioural data variables, we conducted post-hoc analyses of variance, separately for each visit entering site as factor, or chi square analyses for dichotomous variables. Analyses were performed in SPSS v25.

To assess for the effects of site on neuroimaging data, we conducted post-hoc analyses of variance, entering site as factor. We used the MarsBar tool (Brett, et al., 2002) to extract RoI data for the listed regions and then exported these to SPSS for further analyses.

*Demographic and Behavioural Results: Site Differences*

There are no significant differences between sites for age (F(4,31) = 1.58, p = 0.21), sex (χ^2^ = 7.14, p = 0.13), handedness (χ^2^ = 1.78, p = 0.78), or education [(χ^2^ = 24.53, p = 0.65)].

At week 0 there were no significant site differences for total correct (all) faces (F(4,31) = 1.55, p = 0.21), correct congruent faces (F(4,31) = 1.62, p = 0.19), correct incongruent faces (F(4,31) = 1.47, p = 0.24), overall accuracy (F(4,31) = 2.02, p = 0.12), accuracy for incongruent trials (F(4,31) = 1.1, p = 0.38), accuracy for happy faces (F(4,31) = 1.87, p = 0.14) or accuracy for fear faces (F(4,31) = 1.91, p = 0.13). In addition, there were no significant site differences for RT on total correct trials (F(4,31) = 0.48, p = 0.75), for RT on congruent trials (F(4,31) = 0.69, p = 0.6), for RT on incongruent trials (F(4,31) = 0.37, p = 0.83), for RT on happy face trials (F(4,31) = 0.3, p = 0.88) or RT on fear face trials (F(4,31) = 0.81, p = 0.53).

However, there was a significant differences for total errors (F(4,31) = 2.84, p = 0.041), which was driven by significant differences between MCU (mean = 14.0, standard deviation = 6.98) and QNS (mean = 3.67, standard deviation = 3.2). In addition, there was a significant difference for accuracy for congruent trials (F(4,31) = 2.84, p = 0.041), but post-hoc analyses revealed no significant differences between sites.

At week 2, there were no significant site differences for total correct (all) faces (F(4,31) = 0.95, p = 0.46), correct congruent faces (F(4,31) = 0.94, p = 0.46), correct incongruent faces (F(4,31) = 0.86, p = 0.5), total errors (F(4,31) = 2.13, p = 0.1), overall accuracy (F(4,31) = 2.13, p = 0.1), accuracy for congruent trials (F(4,31) = 2.18, p = 0.09), accuracy for incongruent trials (F(4,31) = 1.7, p = 0.18), or accuracy for happy face trials (F(4,31) = 1.24, p = 0.32). However, there was a significant site difference for accuracy on fear face trials (F(4,31) = 2.81, p = 0.042), which was driven by significant differences between MCU (mean = 0.87, standard deviation = 0.07) and QNS (mean = 0.98, standard deviation = 0.02).

Regarding RT, there were no significant site differences on total correct trials (F(4,31) = 1.15, p = 0.35), on congruent trials (F(4,31) = 1.03, p = 0.4), on incongruent trials (F(4,31) = 1.27, p = 0.3), on happy face trials (F(4,31) = 1.06, p = 0.39) or on fear face trials (F(4,31) = 1.23, p = 0.32).

At week 8, there were no significant site differences for total correct (all) faces (F(4,31) = 0.82, p = 0.52), correct congruent faces (F(4,31) = 0.92, p = 0.47), correct incongruent faces (F(4,31) = 0.69, p = 0.6), total errors (F(4,31) = 0.29, p = 0.88), overall accuracy (F(4,31) = 0.55, p = 0.7), accuracy for congruent trials (F(4,31) = 0.65, p = 0.63), accuracy for incongruent trials (F(4,31) = 0.34, p = 0.85), accuracy for happy face trials (F(4,31) = 0.86, p = 0.5) or accuracy for fear face trials (F(4,31) = 0.47, p = 0.76). In addition, there were no significant site differences for RT on total correct trials (F(4,31) = 1.24, p = 0.32), on congruent trials (F(4,31) = 1.14, p = 0.36), on incongruent trials (F(4,31) = 1.37, p = 0.27), on happy face trials (F(4,31) = 1.2, p = 0.33) or on fear face trials (F(4,31) = 1.29, p = 0.29).

*Neuroimaging data – Test-Retest Reliability from week 0 to week 2*

1. *All faces trials*

Results when comparing only the first two time-points showed similar results to those when comparing all three time-points. The voxel-wise ICC estimates were moderate, at best, for regions showing significant BOLD activation (*p* _FWE-corrected_ = 0.05) for the *‘all faces trials’* condition (see table S1). We observed moderate reliability (median ICC > 0.5) within visual regions, including left FFG, but also in the left insula. The left medial frontal gyrus and the left insula, the right superior parietal lobule and the right cuneus showed modest ICC values of > 0.4, but < 0.5. The right thalamus, the right superior temporal gyrus and the right middle frontal gyrus had poor ICC values (ICC ≤ 0.4).

1. *Congruent faces trials*

Significant activation (*p* _FWE-corrected_ = 0.05) was observed in response to for *congruent faces* in the right fusiform gyrus, the right precuneus, the left post-central and middle temporal gyri and also the insula (bilaterally), the right pre-central and middle frontal gyri and the left inferior frontal gyrus (see Table S1). Neural activation within these regions showed modest to moderate reliability, with median ICCs >0.4 but <0.6. Additionally, significant activation (*p* _FWE-corrected_ = 0.05) was observed within the left medial frontal gyrus, the left claustrum and the right middle frontal gyrus, but reliability in these regions was low, ICCs <0.4. The right thalamus, the right post-central gyrus and the right insula as well as the left parahippocampal gyrus had ICCs <0.3, reflective of poor reliability.

1. *Incongruent Faces*

The right precuneus and superior parietal lobule and the left insula had significant activation (*p* _FWE-corrected_ = 0.05) for *incongruent faces* (Table S1). Reliability was moderate in these regions, with median ICCs = 0.5, (0.51-0.53). Significant activation was also apparent in the superior temporal gyrus (bilaterally), the right middle frontal gyrus, the left middle temporal gyrus, the right post-central gyrus, but ICC values for these regions were modest, ICCs >0.3 but <0.5 (0.34-0.47). Poor reliability was observed within the right thalamus, the right post-central gyrus, the left posterior cingulate and the right inferior parietal lobule, median ICC < 0.3.

1. *Fear minus Happy Words and Faces*

The voxel-wise ICC estimates for more cognitively demanding comparisons, such as *fear minus happy words* contrasts were very poor (median ICC = 0.1; Table 5). For the *fear word minus happy word* contrast**,** significant activation (*p* _FWE-corrected_ = 0.05) was observed within the right supramarginal gyrus and the right inferior parietal lobule. For the *fear face minus happy face* contrasts, significant activation (*p* _FWE-corrected_ = 0.05) was observed within right superior temporal gyri, ICC = 0.26.

1. *Incongruent minus Congruent* *trials* contrast

For the *incongruent minus congruent* contrast, significant activation (*p* _FWE-corrected_ = 0.05) was observed for the precuneus and cingulate, bilaterally, as well as within the left middle and inferior frontal gyri, the left pre-central gyrus, the left paracentral lobule, middle temporal gyrus and anterior cingulate and the right angular and inferior frontal gyri . Median ICC values within these regions were < 0.25 (Table S1).

1. *iI minus cI contrast*

For the *iI* (an incongruent trial preceded by an incongruent trial) *minus cI* (incongruent trial preceded by a congruent trial) contrast, we observed significant activation, *p* _uncorrected_ = 0.001 within bilateral cingulate gyri and the hippocampus. However, median ICC values were poor.

--- *table Supplementary Table 1 about here* ---

*Neuroimaging data: Site differences*

For the test-retest analyses of the week 0, 2 and 8 data there were three RoIs (right superior temporal gyrus BA22; left insula BA13; left cingulate gyrus BA24) for which a significant main effect of site was observed. However, the site by time interactions were non-significant. For these, the SIEMENS scanner performed differently than the GE scanners, but, in addition, for the right superior temporal gyrus BA22 the GE Signa and GE Discovery also showed differences.

For the test-retest analyses of the week 0 to week 2 data there was one RoI (left parietal lobe, precentral gyrus) for which there was a significant main effect of site, with the SIEMENS scanner performing differently to the GE scanners. However, the site by time interaction was non-significant.

***Supplementary Table 1.*** *Neuroimaging data on all contrasts conducted for the first two time-points*

| **K** | | **FWE corrected p** | **Region** | | | | | **t** | **x** | **y** | **z** | | **Median ICC** |
| --- | --- | --- | --- | --- | --- | --- | --- | --- | --- | --- | --- | --- | --- |
| **All faces** | | | | | | | | | | | | |  |
| 12758 | | 0.000 | Right | | Occipital Lobe | FFG | BA37 | 14.32 | 38 | -50 | -18 | | 0.54 |
| 6028 | | 0.000 | Left | | Frontal Lobe | Medial Frontal Gyrus | BA6 | 13.31 | -2 | -2 | 56 | | 0.47 |
| 1992 | | 0.000 | Right | |  | Thalamus |  | 12.02 | 22 | -30 | -4 | | 0.25 |
| 2304 | | 0.000 | Right | |  | Insula | BA13 | 11.27 | 34 | 26 | 0 | | 0.39 |
| 450 | | 0.000 | Right | | Parietal Lobe | Superior Parietal Lobule | BA7 | 11.02 | 30 | -52 | 44 | | 0.41 |
| 485 | | 0.000 | Left | |  | Insula | BA13 | 10.46 | -34 | 18 | 4 | | 0.52 |
| 57 | | 0.000 | Left | |  | Insula | BA13 | 9.62 | -50 | -40 | 20 | | 0.43 |
| 331 | | 0.000 | Right | |  | Insula | BA22 | 8.46 | 46 | -30 | -4 | | 0.31 |
| 84 | | 0.000 | Right | | Frontal Lobe | Middle Frontal Gyrus | BA9 | 8.17 | 30 | 42 | 20 | | 0.21 |
| 32744 | | 0.000 | Right | | Posterior Lobe | Declive |  | 17.16 | 14 | -82 | -12 | | 0.56 |
| 3447 | | 0.000 | Right | | Frontal Lobe | Middle Frontal Gyrus | BA46 | 10.88 | 46 | 34 | 16 | | 0.39 |
| 585 | | 0.000 | Right | | Temporal Lobe | Superior Temporal Gyrus | BA22 | 8.9 | 60 | -42 | 12 | | 0.39 |
| 80 | | 0.000 | Right | | Parietal Lobe | Post-central Gyrus | BA43 | 8.24 | 58 | -14 | 16 | | 0.10 |
| 188 | | 0.000 | Right | | Occipital Lobe | Cuneus | BA7 | 8.16 | 14 | -70 | 40 | | 0.48 |
| 58 | | 0.000 | Left | | Limbic Lobe | Parahippocampal Gyrus; Amygdala |  | 8.0 | -20 | -6 | -16 | | 0.06 |
|  | |  |  | |  |  |  |  |  |  |  | |  |
| **Congruent** | | | | | | | | | | | | | |
| 13575 | | 0.000 | Right | | Occipital Lobe | Middle Occipital Lobe | BA18 | 15.64 | 40 | -86 | -6 | | 0.54 |
| 432 | | 0.000 | Right | |  | Thalamus |  | 12.8 | 22 | -30 | -4 | | 0.2 |
| 453 | | 0.000 | Right | | Parietal Lobe | Precuneus | BA7 | 12.44 | 30 | -50 | 44 | | 0.47 |
| 1805 | | 0.000 | Left | | Frontal Lobe | Medial Frontal Gyrus | BA6 | 11.8 | -2 | -4 | 50 | | 0.37 |
| 718 | | 0.000 | Left | | Limbic Lobe | Parahippocampal Gyrus | BA28 | 11.64 | -22 | -30 | -8 | | 0.22 |
| 4112 | | 0.000 | Left | | Parietal Lobe | Post-central Gyrus | BA2 | 10.48 | -54 | -20 | 40 | | 0.47 |
| 71 | | 0.000 | Left | | Sub-lobar | Insula | BA13 | 10.11 | -50 | -40 | 20 | | 0.45 |
| 347 | | 0.000 | Right | | Frontal Lobe | Pre-central Gyrus | BA4 | 8.74 | 46 | -6 | 54 | | 0.41 |
| 58 | | 0.000 | Right | | Parietal Lobe | Post-central Gyrus | BA43 | 8.67 | 58 | -14 | 16 | | 0.25 |
| 29 | | 0.000 | Left | | Temporal Lobe | Middle Temporal Gyrus | BA22 | 7.74 | -50 | -44 | 4 | | 0.42 |
| 43 | | 0.001 | Right | | Frontal Lobe | Medial Frontal Gyrus | BA9 | 7.56 | 28 | 44 | 20 | | 0.07 |
| 123 | | 0.001 | Right | | Sub-lobar | Insula | BA22 | 7.52 | 48 | -26 | -2 | | 0.19 |
| *96* | | *0.002* | *Right* | | *Frontal Lobe* | *Middle Frontal Gyrus* | *BA9* | *7.23* | *48* | *26* | *24* | | *0.46* |
| *58* | | *0.002* | *Right* | | *Frontal Lobe* | *Middle Frontal Gyrus* | *BA6* | *7.15* | *30* | *-4* | *46* | | *0.3* |
| *22* | | *0.002* | *Right* | | *Sub-lobar* | *Insula* | *BA13* | *7.13* | *44* | *0* | *4* | | *0.44* |
| *52* | | *0.005* | *Left* | | *Frontal Lobe* | *Inferior Frontal Gyrus* | *BA9* | *6.85* | *-52* | *14* | *22* | | *0.41* |
|  | |  |  | |  |  |  |  |  |  |  | |  |
| **Incongruent** | | | | | | | | | | | | | |
| 1841 | | 0.000 | Right | | Sub-lobar | Thalamus |  | 13.01 | 20 | -30 | -4 | | 0.26 |
| 678 | | 0.000 | Right | | Parietal Lobe | Superior Parietal Lobule | BA7 | 12.39 | 32 | -54 | 48 | | 0.51 |
| 5828 | | 0.000 | Left | | Parietal Lobe | Post-central Gyrus | BA2 | 11.07 | -54 | -22 | 40 | | 0.5 |
| 2622 | | 0.000 | Right | | Frontal Lobe | Middle Frontal Gyrus | BA36 | 9.71 | 48 | 30 | 14 | | 0.39 |
| 165 | | 0.000 | Left | | Temporal Lobe | Middle Temporal Lobe | BA22 | 9.65 | -50 | -44 | 4 | | 0.43 |
| 87 | | 0.000 | Right | | Parietal Lobe | Post-central Gyrus | BA43 | 9.41 | 56 | -14 | 20 | | 0.14 |
| 349 | | 0.000 | Right | | Temporal Lobe | Superior Temporal Gyrus | BA22 | 8.88 | 62 | -38 | 12 | | 0.38 |
| 62 | | 0.000 | Left | | Sub-lobar | Insula | BA13 | 8.8 | -50 | -40 | 20 | | 0.54 |
| 74 | | 0.000 | Right | | Parietal Lobe | Post-central Gyrus | BA2 | 8.13 | 54 | -20 | 38 | | 0.34 |
| 95 | | 0.000 | Right | | Occipital Lobe | Precuneus | BA31 | 7.84 | 22 | -70 | 34 | | 0.53 |
| *44* | | *0.003* | *Left* | | *Limbic Lobe* | *Posterior Cingulate* | *BA23* | *7.04* | *2* | *-30* | *23* | | *0.15* |
| 23 | | 0.01 | Right | | Parietal Lobe | Inferior Parietal Lobe | BA40 | 6.58 | 50 | -38 | 44 | | 0.27 |
|  | |  |  | |  |  |  |  |  |  |  | |  |
| **Incongruent minus Congruent**  **p(uncorrected)** | | | | | | | | | | | | | |
| 276 | | 0.000 | Right | | Parietal Lobe | Angular Gyrus | BA39 | 5.5 | 40 | -56 | 46 | | 0.24 |
| 158 | | 0.001 | Left | | Frontal Lobe | Pre-central Gyrus | BA6 | 5.23 | -12 | -14 | 70 | | -0.05 |
| 148 | | 0.002 | Left | | Parietal Lobe | Precuneus | BA7 | 5.14 | -22 | -64 | 42 | | 0.14 |
| 1550 | | 0.000 | Right | | Frontal Lobe | Inferior Frontal Gyrus | BA45 | 5.13 | 58 | 18 | 16 | | 0.19 |
| 365 | | 0.000 | Left | | Limbic Lobe | Cingulate Gyrus | BA32 | 5.06 | -10 | 14 | 38 | | 0.2 |
| 150 | | 0.001 | Left | | Frontal Lobe | Inferior Frontal Gyrus | BA45 | 5.06 | -40 | 30 | -2 | | 0.16 |
| 455 | | 0.000 | Left | | Frontal Lobe | Pre-central Gyrus | BA6 | 4.96 | -42 | -8 | 42 | | 0.24 |
| 81 | | 0.013 | Left | | Frontal Lobe | Middle Frontal Gyrus | BA6 | 4.89 | -30 | 0 | 46 | | 0.18 |
| 65 | | 0.024 | Left | | Frontal Lobe | Paracentral Lobule | BA5 | 4.69 | -18 | -38 | 58 | | 0.14 |
| 113 | | 0.004 | Left | | Temporal Lobe | Middle Temporal Gyrus |  | 4.65 | -62 | -28 | 2 | | 0.2 |
| 69 | | 0.02 | Left | | Limbic Lobe | Anterior Cingulate | BA24 | 4.29 | 2 | 34 | 18 | | 0.16 |
|  | |  |  | |  |  |  |  |  |  |  | |  |
| **Word: Fear minus Happy** | | | | | | | | | | | | | |
| *154* | | *0.014* | *Right* | | *Temporal Lobe* | *Supramarginal Gyrus* | *BA40* | *4.8* | *64* | *-48* | *30* | | *-0.1* |
|  | |  | *Right* | | *Parietal Lobe* | *Inferior Parietal Lobule* | *BA40* | *4.46* | *62* | *-40* | *42* | | *0* |
|  | |  |  | |  |  |  |  |  |  |  | |  |
| **Face: Fear minus Happy** | | | | | | | | | | | | | |
| *162* | | *0.011* | *Right* | | *Temporal Lobe* | *Superior Temporal Gyrus* | *BA41* | *5.47* | *48* | *-38* | *2* | | *0.26* |
|  | |  |  | |  |  |  |  |  |  |  | |  |
| **iI minus cI** | | | | | | | | | | | | | |
| **K** | | **peak p(unc)** | | **Region** | | | | **t** | **x** | **y** | **z** | **Median ICC** | |
| 8 | | 0.000 | Right | | Limbic Lobe | Cingulate Gyrus | BA31 | 4.01 | 24 | -28 | 36 | | 0 |
| 3 | | 0.000 | Left | | Limbic Lobe | Cingulate Gyrus | BA24 | 3.75 | -16 | 8 | 42 | | 0 |
| 4 | | 0.001 | Left | | Temporal Lobe | Hippocampus |  | 3.53 | -30 | -48 | 6 | | 0 |
|  | |  |  | |  |  |  |  |  |  |  | |  |

K = cluster extent; FWE = Family-wise-error; BA = Brodman Area; unc = uncorrected; iI = incongruent trial preceded by an incongruent trial; cI = congruent trial preceded by an incongruent trial. ***italics*: FWE-corrected p-value = 0.05; for iI minus cI and incongruent minus congruent**

**results from an uncorrected threshold, p uncorrected = 0.001 are shown**
